# Phenolic Content and Antimicrobial and Anti-Inflammatory Effects of *Solidago virga-aurea*, *Phyllanthus niruri*, *Epilobium angustifolium*, *Peumus boldus*, and *Ononis spinosa* Extracts

**DOI:** 10.3390/antibiotics9110783

**Published:** 2020-11-06

**Authors:** Claudio Ferrante, Annalisa Chiavaroli, Paola Angelini, Roberto Venanzoni, Giancarlo Angeles Flores, Luigi Brunetti, Massimiliano Petrucci, Matteo Politi, Luigi Menghini, Sheila Leone, Lucia Recinella, Gokhan Zengin, Gunes Ak, Massimo Di Mascio, Francesco Bacchin, Giustino Orlando

**Affiliations:** 1 Department of Pharmacy, Università degli Studi “Gabriele d’Annunzio”, via dei Vestini 31, 66100 Chieti, Italy; claudio.ferrante@unich.it (C.F.); annalisa.chiavaroli@unich.it (A.C.); luigi.brunetti@unich.it (L.B.); matteo.politi@unich.it (M.P.); luigi.menghini@unich.it (L.M.); sheila.leone@unich.it (S.L.); lucia.recinella@unich.it (L.R.); giustino.orlando@unich.it (G.O.); 2 Department of Chemistry, Biology and Biotechnology, University of Perugia, 06100 Perugia, Italy; roberto.venanzoni@unipg.it (R.V.); giancarlo.angelesflores@studenti.unipg.it (G.A.F.); 3 Omega Pharma Srl, Via Milano 129, 22063 Cantù (CO), Italy; m.petrucci@omegapharmasrl.com; 4 Department of Biology, Science Faculty, Selcuk Universtiy, Campus, Konya, 42130 Konya, Turkey; akguneselcuk@gmail.com; 5 Veridia Italia Srl, via Raiale 285, 65100 Pescara, Italy; massimo.dimascio96@gmail.com (M.D.M.); f.bacchin@veridia.it (F.B.)

**Keywords:** *Solidago virga-aurea*, *Phyllanthus niruri*, *Epilobium angustifolium*, *Peumus boldus*, *Ononis spinosa*, antimicrobial, antioxidant/anti-inflammatory, bioinformatics

## Abstract

Prostatitis is an inflammatory condition that is related to multiple infectious agents, including bacteria and fungi. Traditional herbal extracts proved efficacious in controlling clinical symptoms associated with prostatitis. In this context, the aim of the present study was to explore the efficacy of extracts from *Solidago virga-aurea*, *Ononis spinosa*, *Peumus boldus*, *Epilobium angustifolium,* and *Phyllanthus niruri* against bacterial (*Escherichia coli, Pseudomonas aeruginosa, Staphylococcus aureus, Bacillus cereus*) and fungi strains (*Candida albicans; C. tropicalis*) involved in prostatitis. Additionally, anti-mycotic effects were tested against multiple species of dermatophytes (*Trichophyton rubrum, T. tonsurans, T. erinacei, Arthroderma crocatum, A. quadrifidum, A. gypseum, A. currey*, and *A. insingulare*). Antioxidant effects were also evaluated in isolated rat prostates challenged with lipopolysaccharide (LPS), and phytochemical analyses were conducted to identify and quantify selected phenolic compounds, in the extracts. Finally, a bioinformatics analysis was conducted to predict putative human and microbial enzymes targeted by extracts’ phytocompounds and underlying the observed bio-pharmacological effects. The phytochemical analysis highlighted that rutin levels could be crucial for explaining the highest antibacterial activity of *P. boldus* extract, especially against *E. coli* and *B. cereus*. On the other hand, in the *E. angustifolium* extract, catechin concentration could partially explain the highest efficacy of this extract in reducing lipid peroxidation, in isolated rat prostates stimulated with LPS. Concluding, the results of the present study showed moderate antimicrobial and anti-inflammatory effects induced by water extracts of *S. virga-aurea, P. boldus, E. angustifolium, P. niruri*, and *O. spinosa* that could be related, at least partially, to the phenolic composition of the phytocomplex.

## 1. Introduction

Prostatitis is an inflammatory condition that is related to multiple infectious agents, including bacteria and fungi [1,2,3]. The inflammatory pathways underlying prostatitis have been increasingly studied in recent years, and herbal extracts, especially in combination, have been revealed as a promising tool for the management of clinical symptoms related to the burden of inflammation/oxidative stress occurring in prostatitis [4]. Strictly related to this disorder, inflammation of the lower urinary tract (LUTS) is reported as the second most common human infectious disorder [5]. Currently, α-blockers represent the first-choice treatment for LUTS in patients diagnosed with both prostatitis and benign prostate hyperplasia (BPH) [6]. On the other hand, traditional herbal extracts were efficacious in controlling the inflammatory component associated with these disorders [7,8,9,10], with the efficacy that could be improved through their pharmacological associations [11]. This is consistent, at least in part, with the scientific literature suggesting the benefits of the combination of herbal extracts, usually employed as food supplements, for treating chronic inflammatory diseases [12,13,14,15,16]. Increasing attention to alternative medications, including nutraceuticals and herbal preparations that could display a lower grade of side effects, has led to the exploration of extracts prepared from plants traditionally used by folk populations to manage inflammatory conditions [14]. These extracts, especially those prepared via traditional infusions or decoctions, could not only be effective and safe due to their long-term use, but also represent novel strategies for implementing local botanical chain productions [15,16].

*Phyllanthus niruri* is a traditional Ayurvedic remedy that has long been employed in a wide range of inflammatory disorders. It is also able to induce anti-proliferative effects on human prostate cancer PC3 cell lines [17]. Boldine, the main active principle of *Peumus boldus*, revealed to be effective in inducing apoptosis in human bladder cancer cells, as well [18]. *Ononis spinosa* is a folk remedy used in the treatment of urinary tract infections and stones [19]. Analogous traditional uses have been described for *Solidago virga-aurea*, although the scientific literature reported anti-proliferative effects, possibly related to the presence of terpene and phenolic compounds, in the phytocomplex [20,21]. Similarly, *Epilobium angustifolium* has been shown to be effective in preventing BPH [22] due to its richness in phenolic compounds [23].

In this context, the aim of the present study was to explore the antimicrobial and anti-inflammatory effects of water extracts from *S. virga-aurea*, *O. spinosa*, *P. boldus*, *E. angustifolium*, and *P. niruri.* Although the water solubility of plant secondary metabolites could be limited, previous studies of ours suggested the use of microwave- and ultrasound-assisted methods for improving phenol extraction that was paralleled by increased anti-inflammatory, antioxidant, and antimicrobial effects [16,24,25]. In the present study, the extracts’ antimicrobial activity was tested against bacterial (*Escherichia coli, Pseudomonas aeruginosa, Staphylococcus aureus*, and *Bacillus cereus*) and fungi strains (*Candida albicans, C. tropicalis*) involved in prostatitis and LUTS [2,3,26,27,28]. Additionally, anti-mycotic activities were also tested against multiple species of dermatophytes (*Trichophyton rubrum, T. tonsurans, T. erinacei, Arthroderma crocatum, A. quadrifidum, A. gypseum, A. Currey*, and *A. insingulare*). This is consistent with the incidence of dermatophyte infections in patients suffering from urinary tract disorders, including renal failure and prostate adenocarcinoma [29,30,31]. Anti-inflammatory effects, in terms of reduction of prostaglandin production, were also evaluated in a preclinical model of prostatitis consisting of isolated rat prostate specimens challenged with lipopolysaccharide (LPS). The study also compared the efficacy of single extracts and their pharmacological association (Fluxonorm^®^), and the obtained results supported the rationale for their use to treat clinical symptoms related to prostatitis and LUTS. In order to improve our knowledge about the mechanism of action of the studied herbal extracts, a fingerprint analysis via high-performance liquid chromatography coupled to ultraviolet and mass spectrometry (HPLC-UV-MS) was conducted to identify and quantify selected phenolic compounds, in the phytocomplex. Specifically, gallic acid, rutin, catechin, and epicatechin were selected considering their intrinsic antimicrobial and anti-inflammatory properties [32,33]. Finally, a bioinformatics analysis was also carried out to predict putative human and microbial enzymes targeted by the extracts’ phytocompounds and underlying the observed bio-pharmacological effects.

## 2. Materials and Methods 

### 2.1. Plant Material

Dry extracts of *Phyllanthus niruri* L. (family Phyllanthaceae)*, Ononis spinosa* L. (family Fabaceae), *Solidago virga-aurea* L. (family Asteraceae)*, Peumus boldus* Molina (family Monimiaceae)*, Epilobium angustifolium* L. (family Onagraceae), and the registered trademark formula Fluxonorm^®^ (*O. spinosa*/*S. virga-aurea*/*P. niruri*/*P. boldus/E. angustifolium* 12.5:12.5:18.7:25.0:31.2), were provided as dried materials by OMEGA PHARMA Srl (Cantù, Italy). Before testing, extracts were rehydrated via ultrasound-assisted extraction at 60 °C for one hour. 

### 2.2. Phytochemical Analysis

*P. niruri*, *E. angustifolium, P. boldus, S. virga-aurea*, and *O. spinosa* extracts (10 mg/mL) were analyzed for phenol quantitative determination using a reversed-phase HPLC-UV-MS in gradient elution mode. The separation was conducted within 60 min, starting from the conditions: water/acetonitrile: 93:7, (*v*/*v*). The details about the gradient are listed in Table 1.

The HPLC apparatus consisted of two PU-2080 PLUS chromatographic pumps, a DG-2080-54 line degasser, a mix-2080-32 mixer, a UV-2075 PLUS UV detector, an AS-2057 PLUS autosampler, and CO-2060 PLUS column thermostat, all made by JASCO (Tokyo, Japan). The used mass spectrometer was an Expression-L compact mass spectrometer (Advion, Ithaca, NY, USA). Integration was performed by JASCO ChromNAV2 chromatography software, and the separation was performed by gradient elution on an Agilent (Santa Clara, CA, USA) InfinityLab Poroshell 120 reverse phase column (C18, 150 mm × 4.6 mm ID, 2.7 µm). The analytical conditions for the separation of gallic acid, catechin, epicatechin and rutin were in agreement with the study by Rodríguez-Delgado and colleagues [34]. Extracts were qualitatively analyzed with an Advion Expression-L compact mass spectrometer (MS) in negative ion mode. MS signal identification was realized through comparison with standard solutions and MS spectra present in the recognized Mass Bank Europe database (https://massbank.eu/MassBank/). Quantitative determination of phenolic compounds identified by MS analysis was performed via a UV detector at 240 nm wavelength. Quantification was done through 7 point calibration curves, with linearity coefficients (R^2^) > 0.999, in the concentration range of 2–140 µg/mL. The area under the curve obtained from the HPLC chromatograms was used to quantify analyte concentration in the extracts.

Protonic nuclear magnetic resonance (^1^H-NMR) analysis was conducted with a Varian 300 MHz spectrometer using a standard proton pulse sequence (s2pul). Samples were prepared as follows: 10 mg/mL gallic acid and 50 mg/mL extract were sonicated in CD_3_OD for 30 min at room temperature. Next, 600 µL of each sample was transferred into the NMR tube and analyzed with the following parameters: 1.706 s acquisition time, 4803.1 Hz width, and 64 scans.

### 2.3. Artemia salina Lethality Test

The cytotoxicity limit of the extracts in the range 0.1–20 mg/mL was evaluated through a lethality bioassay of the brine shrimp *Artemia salina*, as previously reported [16]. The experiments were carried out in triplicate. 

### 2.4. Cell Cultures and Viability Test

The effects of the extracts (100–500 µg/mL) on myocyte C2C12 and prostate cancer PC3 cell viability were determined through the 3-(4,5-dimethylthiazol-2-yl)-2,5-diphenyltetrazolium bromide (MTT) test. The experimental conditions were fully described in our previous paper [35].

### 2.5. Ex Vivo Pharmacological Study

The protective effects of the extracts were investigated through an ex vivo model consisting of isolated prostate challenged with *E. coli* lipopolysaccharide (LPS) at 10 µg/mL, as previously reported [9]. Prostates were collected from 24 Sprague Dawley rats (200–250 g). The study was approved by the Local Ethical Committee and the Italian Ministry of Health. The approval number of the study was the following: authorization N. F4738.N.XTQ, delivered for the period 11 November 2018–11 November 2019. During the study, the prostate specimens were separately treated with the extracts of *O. spinosa, S. virga-aurea, P. niruri, P. boldus*, and *E. angustifolium* (100, 100, 150, 200, and 250 µg/mL, respectively) or Fluxonorm^®^ (800 µg/mL). Afterward, the prostate tissue supernatants were assayed, as described below.

### 2.6. 8-Iso-PGF_2α_ and PGE_2_ Radioimmunoassay

The levels of 8-iso-PGF_2α_ and PGE_2_ in the supernatants were determined by radioimmunoassay (RIA). The detailed protocol was described in our previous study [36].

### 2.7. Antibacterial and Antimycotic Activities

The water extracts of *P. niruri, O. spinosa*, *S. virga-aurea*, *P. boldus*, and *E. angustifolium* were assayed for antibacterial activity against the following Gram-negative and Gram-positive strains: *E. coli* (ATCC 10536); *E. coli* (PeruMycA 2); *E. coli* (PeruMycA 3); *P. aeruginosa* (PeruMycA 5); *S. typhy* (PeruMycA 7); *B. cereus* (PeruMycA 4); *B. subtilis* (PeruMycA 6); *S. aureus* (ATCC 6538). Additionally, the same extracts were assayed for antimycotic effects against different *Candida* and dermatophyte species: *C. tropicalis* (DBVPG 6184); *C. albicans* (DBVPG 6379); *C. parapsilosis* (DBVPG 6551); *C. albicans* (DBVPG 6183); *T. mentagrophytes* (CCF 4823); *T. tonsurans* (CCF 4834); *T. rubrum* (CCF 4879); *T. rubrum* (CCF 4933); *A. crocatum* (CCF 5300); *A. quadrifidum* (CCF 5792); *T. erinacei* (CCF 5930); *A. gypseum* (CCF 6261); *A. currey* (CCF 5207); *A. insingulare* (CCF 5417). The antimicrobial activities of the aforementioned extracts were compared to reference drugs. To investigate the extracts effects on bacterial, *Candida*, and dermatophyte growth, the inhibitory activity was compared to ciprofloxacin, fluconazole and griseofulvin, respectively. The detailed protocols of the present antimicrobial assays have been fully described in recently published papers of ours [37,38].

### 2.8. Bioinformatics

Putative targets were identified according to the bioinformatic method recently described by Gu and colleagues [39]. Briefly, microbial and human proteins targeted by *P. niruri*, *O. spinosa*, *S. virga-aurea*, *P. boldus*, and *E. angustifolium* extracts were predicted using the bioinformatics platform STITCH (http://stitch.embl.de/cgi/network.pl). Docking calculations were conducted using AutoDock Vina PyRx 0.8 software. Crystal structures of target proteins were derived from the Protein Data Bank (PDB) with PDB IDs as follows: 1MJT (nitric oxide synthase (NOS) from *S. aureus*), 2FLQ (nitric oxide synthase (NOS) from *B. cereus*), and 1CX2 [cyclooxygenase-2 (COX-2)]. Discovery studio 2020 visualizer was employed to investigate the protein–ligand non-bonding interactions.

### 2.9. Statistical Analysis

The experimental data related to in vitro and ex vivo studies were analyzed through the analysis of variance (ANOVA) followed by the Newman–Keuls post hoc test. GraphPad Prism software was employed for the statistical analysis, while the software G*Power (v3.1.9.4, University of Kiel, Kiel, Germany) was used for calculating the animal number for the experiments, where *p* < 0.05 was considered statistically significant.

## 3. Results and Discussion

**Figure 1:**
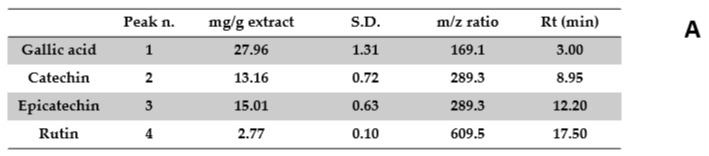

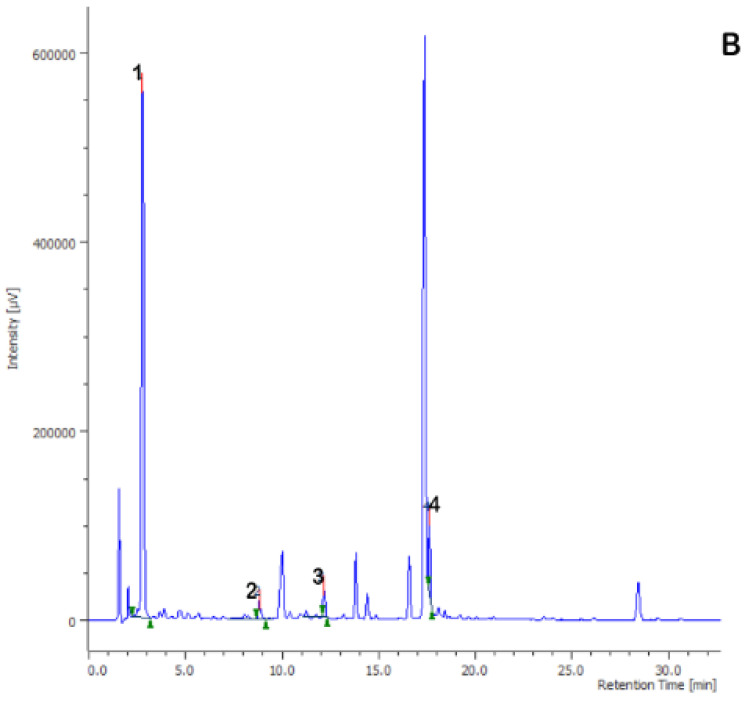
Quantitative analysis of *Phyllanthus niruri* water extract. (**A**): Levels (mg/g extract) of gallic acid (1), catechin (2), epicatechin (3), and rutin (4). (**B**): Chromatogram of selected phenolic compounds.

**Figure 2:**
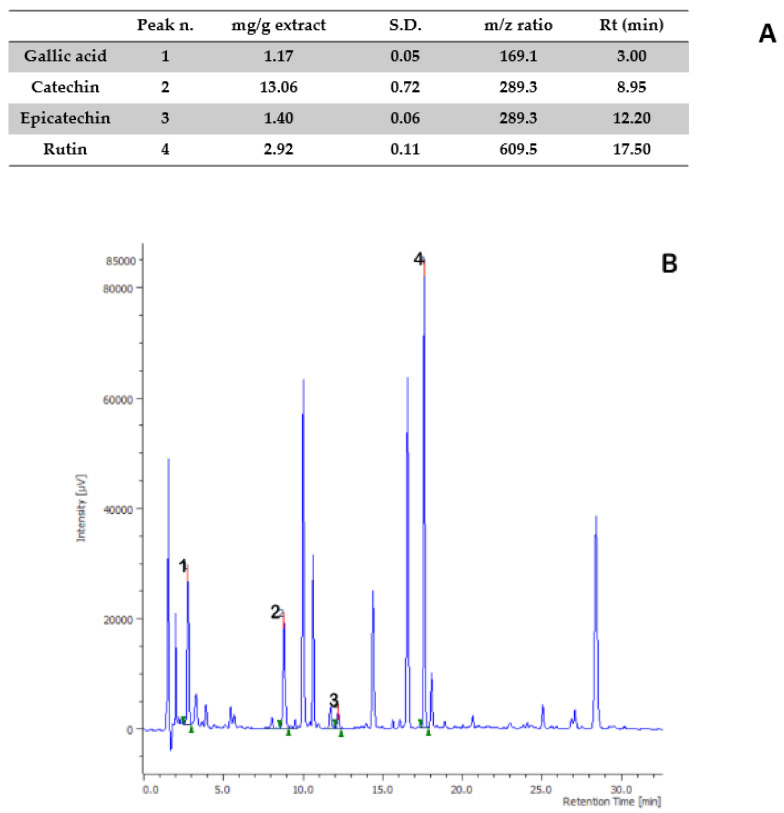
Quantitative analysis of Ononis spinosa water extract. (A): Levels (mg/g extract) of gallic acid (1), catechin (2), epicatechin (3), and rutin (4). (B): Chromatogram of selected phenolic compounds.

**Figure 3:**
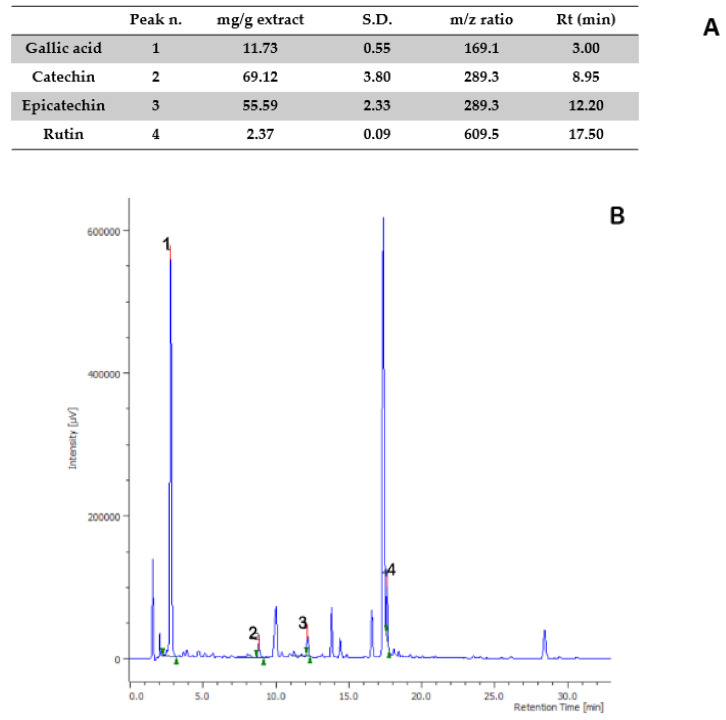
Quantitative analysis of *Epilobium angustifolium* water extract. (**A**): Levels (mg/g extract) of gallic acid (1), catechin (2), epicatechin (3), and rutin (4). (**B**): Chromatogram of selected phenolic compounds.

**Figure 4:**
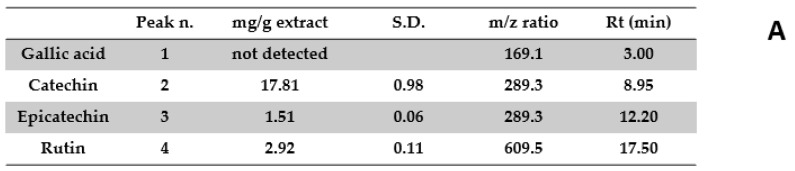

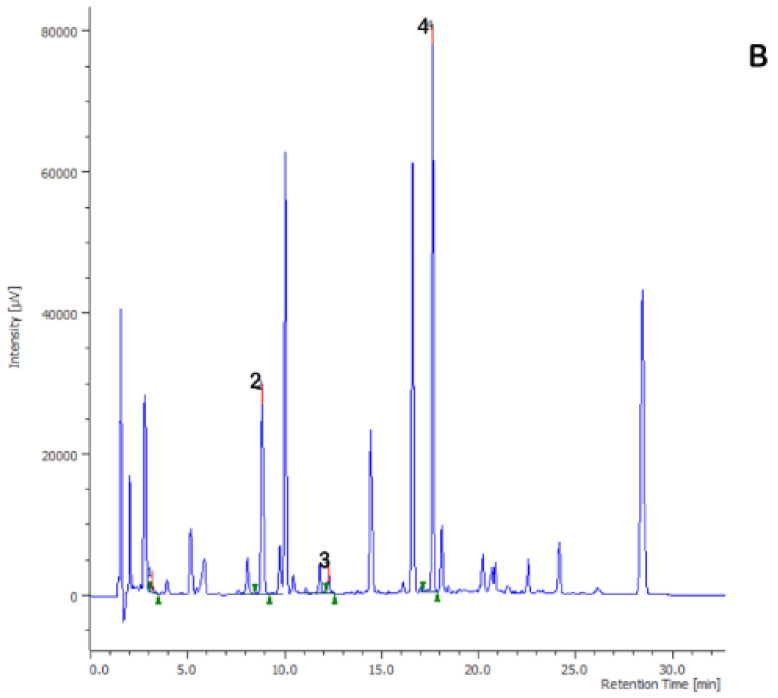
Quantitative analysis of *Solidago virga-aurea* water extract. (**A**): Levels (mg/g extract) of gallic acid (1), catechin (2), epicatechin (3), and rutin (4). (**B**): Chromatogram of selected phenolic compounds.

**Figure 5:**
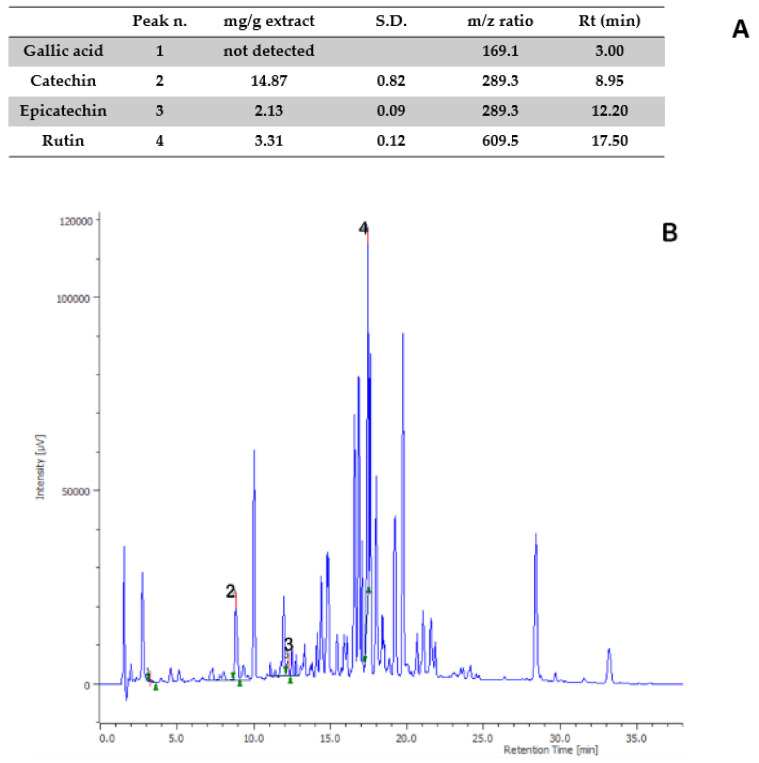
Quantitative analysis of *Peumus boldus* water extract. (**A**): Levels (mg/g extract) of gallic acid (1), catechin (2), epicatechin (3), and rutin (4). (**B**): Chromatogram of selected phenolic compounds.

**Figure 6:**
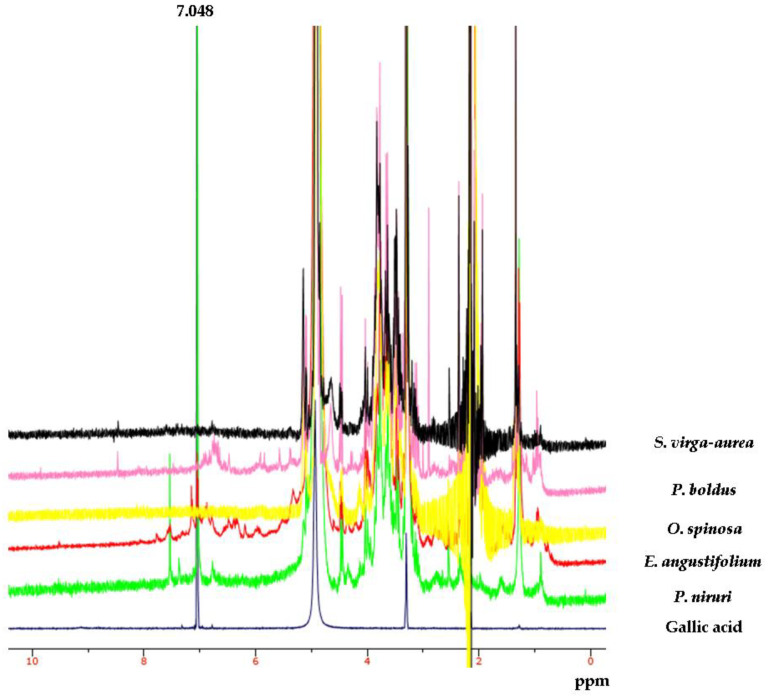
From top to bottom, ^1^H-NMR spectra of *Solidago virga-aurea* (black)*, Peumus boldus* (pink)*, Ononis spinosa* (yellow)*, Epilobium angustifolium* (red), and *Phyllanthus niruri* (green) extracts and gallic acid standard solution (blue). The gallic acid protons signal (H-2, H-6) is visible at 7.048 ppm and is detected in *P. niruri* and *E. angustifolium* extracts.

Aiming to investigate the putative mechanism of action of the tested extracts, HPLC-UV-MS fingerprint analysis was carried out in order to measure the levels of selected phenolic compounds, namely gallic acid, catechin, epicatechin, and rutin, that play a master role in the antioxidant/anti-inflammatory response following herbal extract administration [40]. Specifically, the results of HPLC-UV-MS analysis depicted in Figure 1, Figure 2, Figure 3, Figure 4 and Figure 5 show that the level of gallic acid is higher in *P. niruri* and *E. angustifolium* extracts, whereas *P. boldus*, *O. spinosa* and *S. virga-aurea* do not show a relevant amount of this compound. These data were also consistent with the ^1^H-NMR analyses (Figure 6), where the gallic acid protons signal (H-2, H-6) was detected at 7.048 ppm, mainly in *P. niruri* and *E. angustifolium* extracts, respectively. In the same extracts, the catechin fraction was also present at higher concentrations, compared to *S. virga-aurea*, *P. boldus*, and *O. spinosa* extracts, whereas *P. boldus* extract showed higher rutin concentration. In each figure, the concentration data were substantiated by related UV and MS chromatograms that highlighted a complete separation of the four measured phenolics within a 20 min HPLC run. Specifically, each analyte peak was qualitatively analyzed with MS detector in negative ion mode and the presence of gallic acid (m/z = 169.1; retention time = 3.00 min), catechin (m/z = 289.3; retention time = 8.95 min), epicatechin (m/z = 289.3; retention time = 12.20 min), and rutin (m/z = 609.5; retention time = 17.50 min) was identified through comparison with related standards and with MS data collected by MassBank Europe (https://massbank.eu/MassBank/). According to the quantitative analysis conducted through the UV detector (set at 240 nm wavelength), *E. angustifolium* displayed the richest phenolic profile from both qualitative and quantitative points of view. This was consistent, albeit partially, with literature data suggesting the richness in phenolic compounds of *E. angustifolium* as a key factor influencing its efficacy in BHP [22,23].

**Figure 7:**
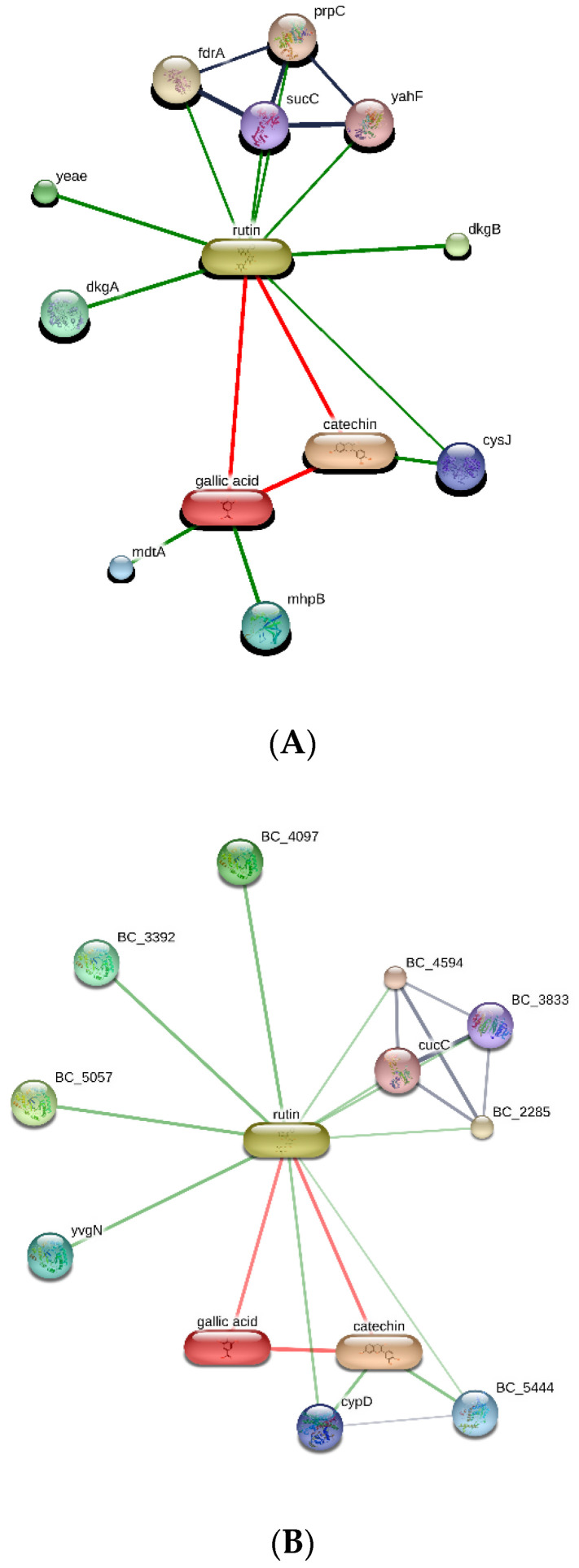
Targets–components analysis conducted on the STITCH bioinformatics platform for unraveling putative interactions between extracts’ phytochemicals and putative proteins involved in the oxidative metabolism of *E. coli* (subfigure (**A**)) and *B. cereus* (subfigure (**B**)). In both subfigures, the prominent position of rutin is highlighted with regards to its putative interactions with bacterial enzymes, among which is *B. cereus* nitric oxide synthase (BC_44).

**Figure 8:**
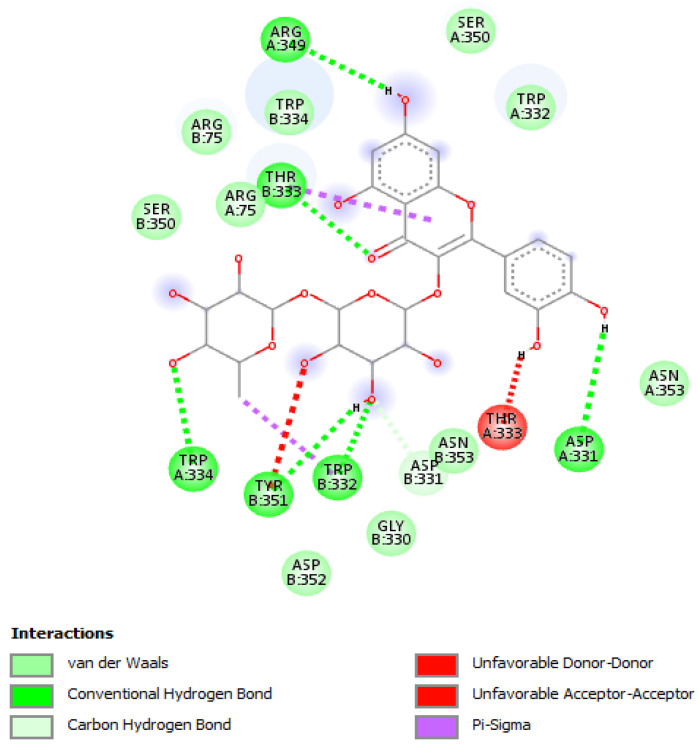
Putative interactions between rutin and *B. cereus* nitric oxide synthase (BC_5444; PDB: 2FLQ). Free energy of binding (ΔG) and affinity (Ki) are −8.9 kcal/mol and 0.3 µM, respectively.

The extracts were also assayed for the brine shrimp (*A. salina*) lethality test to evaluate the biocompatibility limit in the standard range (0.01–10 mg/mL) [41]. The brine shrimp test yielded LC_50_ values >10 mg/mL that were indicatory for choosing a concentration limit at least ten-fold lower than LC_50_ for the following bio-pharmacological tests [16]. In this regard, the extracts were assayed for antimicrobial activity against multiple bacterial and fungi strains (Table 2, Table 3 and Table 4), including *Candida* and dermatophyte species, that could be involved in infectious disorders of the urinary tract [28,29,30,31]. It is of noteworthy interest that most of the tested extracts showed bacteriostatic and mycostatic effects at concentrations much lower than LC_50_ values calculated via the brine shrimp test. In the present multidirectional study, the inhibitory effects of tested extracts on bacterial and fungi strains were expressed as µg/mL in order to better compare the concentrations of the extracts active as antimicrobials with those active as anti-inflammatory/antioxidant agents [16,37,38]. On the other hand, multiple studies suggest the use of arbitrary units to express the antimicrobial activity of herbal extracts as well [42,43,44]. The spectrum of antimicrobial activity seems to be paralleled, at least partially, by the extract qualitative composition. In this regard, *E. angustifolium* was effective against a larger number of microbial species compared to the other tested extracts that displayed lower levels of catechins. The quantitative HPLC analysis also suggested that *E. angustifolium* could have a higher content of total phenols, thus substantiating the wider spectrum of antimycotic activity [16]. Nevertheless, *P. boldus* was the most active as antibacterial agent against both *E. coli* and *B. cereus.* It is sensitive to note that *P. boldus* displayed the highest rutin concentration that could be crucial for the highest antibacterial activity against these strains. This hypothesis seemed to be validated by the bioinformatics analysis conducted through the STITCH platform that highlighted a prominent position for rutin in the scenario of putative interactions between identified phytocompounds and bacterial proteins involved in oxidative metabolism (Figure 7A,B). Regarding the *B. cereus*, a further docking analysis was conducted to calculate the affinity of rutin towards *B. cereus* nitric oxide synthase (BC_44). Intriguingly, the docking run yielded an affinity constant (Ki) in the sub-micromolar range (Figure 8). This result adds to our recent observation about the putative interactions between BC_44 and resveratrol [37]. Nevertheless, the inhibitory effect induced by rutin on *B. cereus* occurred at concentrations higher than 250 µg/mL, thus excluding the interaction of this flavonoid with BC_44 as a key event underlying the antimicrobial effect induced by *P. boldus.* In this regard, we hypothesize that total phenolic compound content could be involved in the antibacterial activity [16], as also suggested by the *P. boldus* HPLC-UV-MS chromatogram and ^1^H-NMR spectrum showing numerous signals related to unidentified phenolic compounds present in this extract.

**Figure 9:**
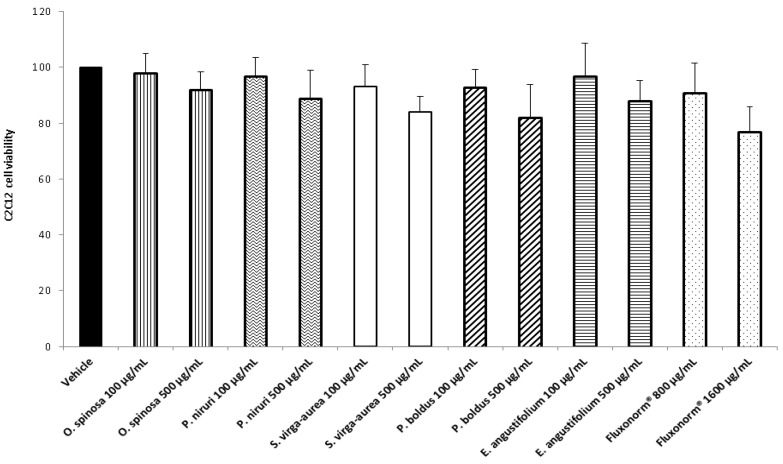
Effect of *Phyllanthus niruri*, *Ononis spinosa*, *Solidago virga-aurea*, *Peumus boldus*, and *Epilobium angustifolium* extracts (100–500 µg/mL) and Fluxonorm^®^ (800–1600 µg/mL) on myocyte C2C12 cell viability (MTT test).

**Figure 10:**
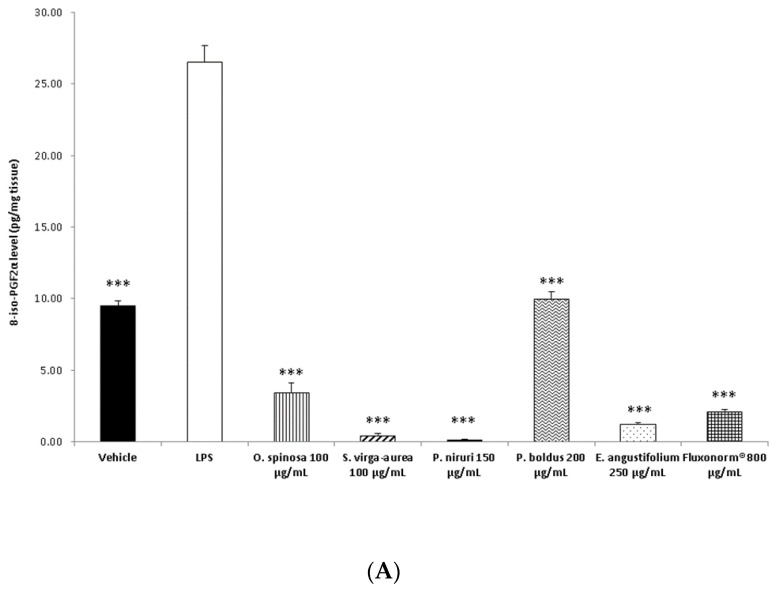

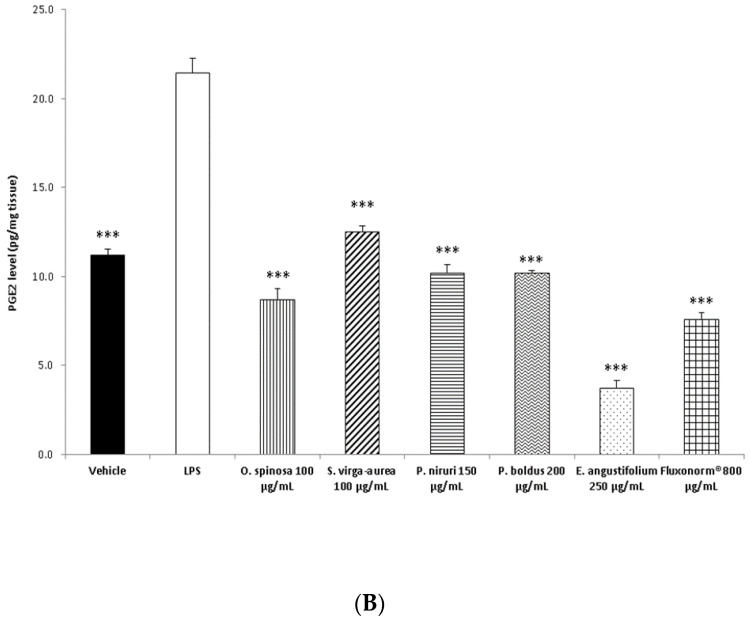
(**A**) Effect of extracts of *Phyllanthus niruri* 100 µg/mL, *Ononis spinosa* 100 µg/mL, *Solidago virga-aurea* 150 µg/mL, *Peumus boldus* 200 µg/mL, and *Epilobium angustifolium* 250 µg/mL water extracts and Fluxonorm^®^ 800µg/mL on 8-iso-prostaglandin (PG)F_2α_ production in isolated rat prostates challenged with *E. coli* lipopolysaccharide (LPS: 10 µg/mL). ANOVA, *p* < 0.0001, post-hoc ***p < 0.001 vs. LPS group. (**B**) Effect of water extracts of *P. niruri* 100 µg/mL, *O. spinosa* 100 µg/mL, *S. virga-aurea* 150 µg/mL, *P. boldus* 200 µg/mL, and *E. angustifolium* 250 µg/mL water extracts and Fluxonorm^®^ 800 µg/mL on prostaglandin (PG)E2 production in isolated rat prostate challenged with *E. coli* lipopolysaccharide (LPS: 10 µg/mL). ANOVA, *p* < 0.0001, post-hoc ***p < 0.001 vs. LPS group.

**Figure 11:**
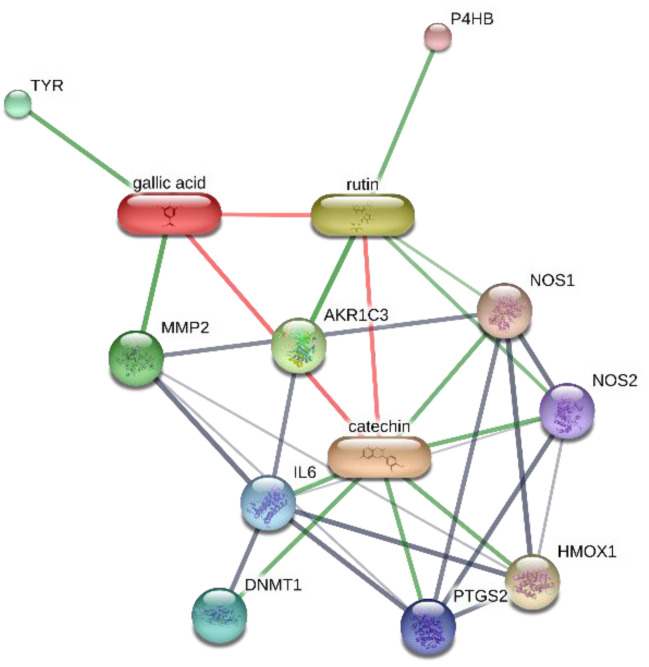
Targets–components analysis related to the putative human proteins that are principally targeted by catechin.

**Figure 12:**
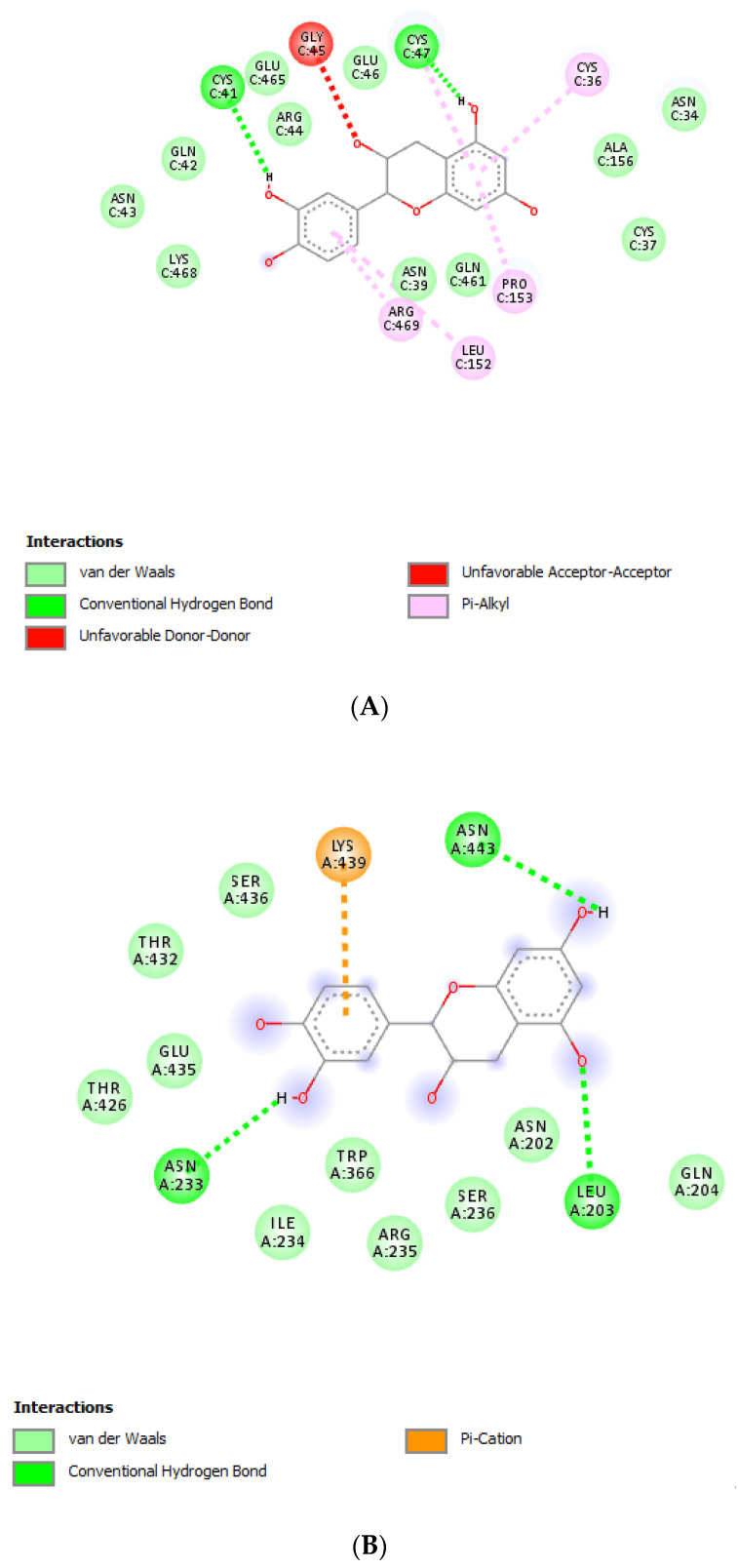
(**A**) Putative interactions between catechin and human cyclooxygenase-2 (COX-2; PDB: 1CX2). Free energy of binding (ΔG) and affinity (Ki) are −9.5 kcal/mol and 0.1 µM, respectively; (**B**) Putative interactions between catechin and human inducible nitric oxide synthase (iNOS; PDB: 1DD7). Free energy of binding (ΔG) and affinity (Ki) are −7.5 kcal/mol and 3.2 µM, respectively.

**Figure 13:**
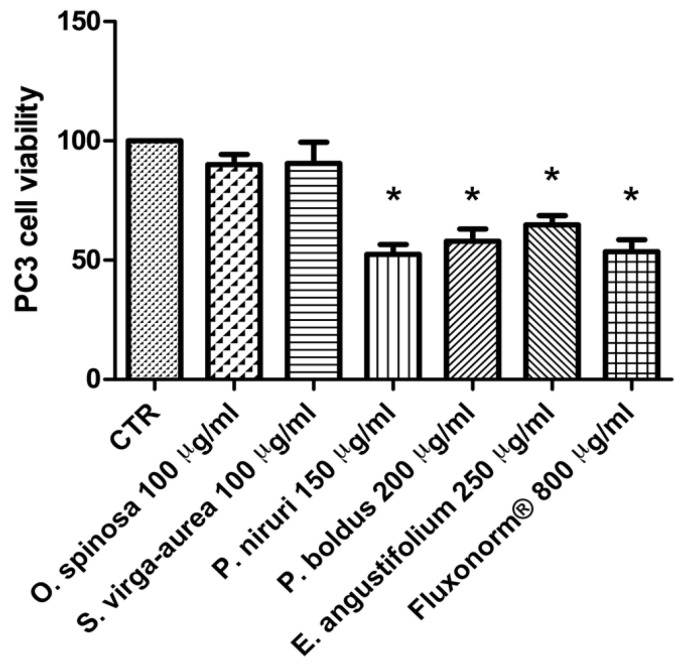
Effect of extracts of *Phyllanthus niruri* 100 µg/mL, *Ononis spinosa* 100 µg/mL, *Solidago virga-aurea* 150 µg/mL, *Peumus boldus* 200 µg/mL, and *Epilobium angustifolium* 250 µg/mL water extracts and Fluxonorm^®^ 800 µg/mL on prostate cancer PC3 cell line viability. ANOVA, *p* < 0.01, post-hoc * *p* < 0.05 vs. CTR group.

The different pattern of antimicrobial activity also supports the rationale for the pharmacological association of tested extracts in order to improve their efficacy. In this regard, in vitro tests were conducted on the non-tumoral cell line C2C12 to expand our comprehension about the intrinsic tolerability and protective effects of the extracts employed as both single treatment and pharmacological associations (Fluxonorm^®^). Specifically, MTT assay was carried out, and extracts were tested in the concentration range 100–500 µg/mL, whereas the Fluxonorm^®^ formula was assayed in the range from 800–1600 µg/mL. The null effect on the cell viability after challenging the cells with the extracts (Figure 9) further confirmed the biocompatibility of the selected water extracts. The pharmacological association Fluxonorm^®^, which was the resulting algebraic sum of the aforementioned extracts used as single ingredients in the ratio reported in Section 2.1, was completely tolerated at the lowest tested concentration (800 µg/mL). By contrast, the cell viability tended to decrease under the biocompatibility limit (80%) at the highest formula concentration (1600 µg/mL). It is rational to hypothesize that the reduced cell viability at the highest tested concentration could be related to the paradoxical phenolic compound-induced oxidative stress that often occurs at elevated concentrations [45,46]. Conversely, under the limit of biocompatibility, the single extracts and Fluxonorm^®^ were able to contrast the burden of oxidative stress and inflammation in isolated rat prostate specimens challenged with LPS, an ex vivo model of prostatitis [11,47]. All extracts were able to completely blunt the LPS-induced level of both PGE_2_ and 8-iso-PGF_2α_ (Figure 10A,B). PGE_2_ is the main prostanoid synthesized by cyclo-oxygenase (COX)-2 in inflammatory conditions [48], and up-regulated levels of PGE_2_ are found in prostate inflammation and cancer [49,50]. While, 8-iso-PGF_2α_ is an isomer of classical prostaglandins that is principally produced by oxidative stress-mediated pathways, including reactive oxygen/nitrogen species (ROS/RNS), that could cause disruptive peroxidation reactions on cellular substrates, such as proteins, lipids and nucleic acids [51]. In this regard, 8-iso-PGF_2α_ deriving from ROS/RNS peroxidation of membrane arachidonic acid represents a diagnostic biomarker for evaluating lipid peroxidation in vivo [52]. On the other hand, a paradoxically reduced level of 8-iso-PGF_2α_ has been observed in superficial bladder cancer, possibly related to the putative vasoconstrictor role of this prostanoid [53]. Currently, the reduced levels of both PGE_2_ and 8-iso-PGF_2α_ following extract treatment are consistent with the qualitative and quantitative profile of the analyzed phytochemicals. However, the best pharmacological profile was showed by *E. angustifolium*, which influenced the antioxidant/anti-inflammatory role of Fluxonorm^®^ the most. This is probably related to its content in catechin and epicatechin, which are known to behave as COX-2 inhibitors [54]. It is sensitive to highlight the clinical study carried out by Micali and colleagues [55], which evidenced a significant reduction in prostate-specific antigen following green tea catechin administration, thus suggesting its potential efficacy in treating/preventing inflammatory prostate diseases. Furthermore, the antiproliferative effects of green tea deriving catechins were demonstrated in multiple cancer cell lines, including prostate cancer LNCaP cells [56]. The pivotal role of catechin in the anti-inflammatory response related to extract treatment was also indicated by bioinformatics analysis (Figure 11). In this regard, besides COX-2, different pro-inflammatory proteins were predicted to be targeted by catechin, including inducible nitric oxide synthase (iNOS). Additionally, the sub-micromolar/micromolar affinity of catechin towards COX-2 and iNOS (Figure 12A,B) agreed with catechin levels in all tested extracts. The highest efficacy of *E. angustifolium* in contrasting the burden of oxidative stress/inflammation in isolate prostate specimens was consistent with the recent findings by Deng and colleagues (2019), as well as the capability of *E. angustifolium* extract to counteract the oxidative stress in male rats subcutaneously injected with testosterone in order to induce BHP [22]. The same authors also suggested that phytocomplex phenolic compounds could be crucial for substantiating the antioxidant/anti-inflammatory effects of *E. angustifolium* in the prostate. Finally, prostate cancer PC3 cells were treated with single extracts and Fluxonorm^®^. Specifically, *E. angustifolium*, *P. niruri*, *P. boldus*, and Fluxonorm^®^ showed similar antiproliferative effects (Figure 13) that could be related, at least partially, to multiple anti-inflammatory and antioxidant mechanisms [57,58,59]. In this context, it is sensitive to hypothesize that phenolic compounds present in the tested extracts could be responsible for the observed antiproliferative effects. However, these effects agree only partially with the levels of selected phenolic compounds measured via HPLC-UV-MS analyses. The chromatograms of the same analyses, but also the NMR spectra, suggest the presence of other unidentified phenolic compounds that could be crucial for the observed bio-pharmacological effects. 

## 4. Conclusions

The results of the present study showed moderate antimicrobial and anti-inflammatory effects induced by water extracts of *S. virga-aurea*, *P. boldus*, *E. angustifolium*, *P. niruri*, and *O. spinosa*, that could be related, at least partially, to the phenolic composition of the phytocomplex. Future phytochemical investigations may further unravel the relationships between extract composition and bio-pharmacological effects.

## Figures and Tables

**Table 1:** Gradient elution.

Time (min)	Flow	%A	%B
0–0.50	1 mL/min	93	7
25		72	28
30		72	28
38		75	25
45		2	98
48		93	7
58		93	7

**Table 2:** Minimal inhibitory concentrations (MICs) of plant extracts against dermatophytes strains.

Dermatophyte Strains (ID)	Minimum Inhibitory Concentration (MIC) *					
	*P. boldus* (µg/mL)	*E. angustifolium* (µg/mL)	*O. spinosa* (µg/mL)	*P. niruri* (µg/mL)	*S. virga-aurea* (µg/mL)	Griseofulvin (µg/mL)
*T. mentagrophytes* (CCF 4823)	78.74 (62.5–125)	99.21 (62.25–125	>250	198.42 (125–250)	>250	2.52 (2–4)
*T. tonsurans* (CCF 4834)	9.84 (7.81–15.62)	12.4 (7.81–15.62)	78.74 (62.5–125)	49.60 (31.25–62.5)	49.6 (31.25–62.5)	0.198 (0.125–0.25)
*T. rubrum* (CCF 4879)	>250	78.74 (62.5–125)	99.21 (62.5–125)	78.74 (62.5–125)	99.21 (62.5–125)	3.175 (2–4)
*T. rubrum* (CCF 4933)	24.80 (15.62–31.25)	24.80 (15.62–31.25)	49.6 (31.25–62.5)	49.6 (31.25–62.5)	99.21 (62.5–125)	1.26 (1–2)
*A. crocatum* (CCF 5300)	19.68 (15.62–31.25)	19.68 (15.62–31.25)	24.80 (15.62–31.25)	39.37 (31.25–62.5)	78.74 (62.5–125)	>8
*A. quadrifidum* (CCF 5792)	39.37 (31.25–62.25)	78.74 (62.5–125)	198.42 (125–250)	>250	198.42 (125–250)	>8
*T. erinacei* (CCF 5930)	>250	198.42 (125–250)	>250	>250	>250	3.174 (2–4)
*A. gypseum* (CCF 6261)	157.49 (125–250)	157.49 (125–250)	>250	>250	>250	1.587 (1–2)
*A. currey* (CCF 5207)	24.80 (15.62–31.25)	39.37 (31.25–62.5)	78.74 (62.5–125)	49.6 (31.25–62.5)	78.74 (62.5–125)	>8
*A. insingulare* (CCF 5417)	39.37 (31.25–62.25)	49.61 (31.25–62.5)	99.21 (62.5–125)	78.74 (62.5–125)	99.21 (62.5–125)	>8

* MIC values are reported as geometric means of three independent replicates (*n* = 3). MIC range concentrations are reported within brackets.

**Table 3:** Minimal inhibitory concentrations (MICs) of plant extracts against yeasts strains.

Yeast Strains (ID)	Minimum Inhibitory Concentration (MIC) *					
	*P. boldus* (µg/mL)	*E. angustifolium* (µg/mL)	*O. spinosa* (µg/mL)	*P. niruri* (µg/mL)	*S. virga-aurea* (µg/mL)	Fluconazole (µg/mL)
*C. tropicalis* (DBVPG 6184)	157.49 (125–250)	49.60 (31.25–62.5)	78.74 (62.5–125)	99.21 (62.5–125)	49.60 (31.25–62.5)	2
*C. albicans* (DBVPG 6379)	>250	198.42 (125–250)	198.42 (125–250)	(≥250)	>250	1
*C. parapsilosis* (DBVPG 6551)	198.42 (125–250)	(≥250)	>250	>250	198.42 (125–250)	4
*C. albicans* (DBVPG 6183)	99.21 (62.5–125)	157.49 (125–250)	>250	>250	>250	2

* MIC values are reported as geometric means of three independent replicates (*n* = 3). MIC range concentrations are reported within brackets.

**Table 4:** Minimal inhibitory concentrations (MICs) of plant extracts against bacterial strains.

Bacterial Strains (ID)	Minimum Inhibitory Concentration (MIC) *					
	*P. boldus* (µg/mL)	*E. angustifolium* (µg/mL)	*O. spinosa* (µg/mL)	*P. niruri* (µg/mL)	*S. virga-aurea* (µg/mL)	Ciprofloxacin (µg/mL)
Gram−						
*E. coli* (ATCC 10536)	24.80 (7.81–15.625)	78.74 (62.5–125)	49.60 (31.25–62.5)	39.37 (31.25–62.5)	78.74 (62.5–125)	<0.12
*E. coli* (PeruMycA 2)	39.37 (31.25–62.5)	157.49 (125–250)	99.21 (62.5–125)	49.60 (31.25–62.5)	49.60 (31.25–62.5)	1.23 (1.95–0.98)
*E. coli* (PeruMycA 3)	99.21 (62.5–125)	198.42 (125–250)	157.49 (125–250)	(≥250)	>250	0.62 (0.98–0.49)
*P. aeruginosa* (PeruMycA 5)	78.74 (62.5–125)	99.21 (62.5–125)	49.60 (31.25–62.5)	24.8 (15.62–31.25)	39.37 (31.25–62.5)	1.23 (1.95–0.98)
*S. typhy* (PeruMycA 7)	157.49 (125–250)	198.42 (125–250)	(≥250)	198.42 (125–250)	(≥250)	0.38 (0.49–0.24)
Gram+						
*B. cereus* (PeruMycA 4)	78.74 (62.5–125)	99.21 (62.5–125)	198.42 (125–250)	49.60 (31.25–62.5)	78.74 (62.5–125)	<0.12
*B. subtilis* (PeruMycA 6)	157.49 (125–250)	198.42 (125–250)	(≥250)	99.21 (62.5–125)	157.49 (125–250)	<0.12
*S. aureus* (ATCC 6538)	198.42 (125–250)	198.42 (125–250)	99.21 (62.5–125)	78.74 (62.5–125)	99.21 (62.5–125)	0.62 (0.98–0.49)

* MIC values are reported as geometric means of three independent replicates (*n* = 3). MIC range concentrations are reported within brackets.
